# Short-term psychodynamic psychotherapy for social anxiety disorder: a meta-analysis of randomized controlled trials

**DOI:** 10.1186/s40359-026-04306-x

**Published:** 2026-03-05

**Authors:** Maud Norén, Karin Lindqvist, Peter Lilliengren, Jakob Mechler

**Affiliations:** 1 Department of Psychology, Uppsala University, Uppsala, Sweden; 2 Department of Psychology, Stockholm University, Stockholm, Sweden; 3 The Erica foundation, Stockholm, Sweden

**Keywords:** Social anxiety, Psychodynamic therapy, STPP efficacy, Meta-analysis, Systematic review

## Abstract

**Background:**

Although established treatments for social anxiety disorder (SAD) are effective, a substantial proportion of patients does not respond. This meta-analysis evaluated the efficacy of psychodynamic therapy (PDT) by assessing its short- and long-term effects on social anxiety symptoms, as well as on secondary measures of depressive symptoms.

**Methods:**

A systematic literature search following PICO criteria identified randomized controlled trials (RCTs) of PDT for SAD published since 1980. Study quality was assessed using the Randomized Controlled Trial Psychotherapy Quality Rating Scale and the Cochrane Risk of Bias tool. At treatment termination, between-group effect sizes were pooled using random effect models. Long-term effects were explored using between- and within-group analyses, depending on available follow-up data.

**Results:**

Eleven eligible RCTs (*n* = 1167), evaluating short-term psychodynamic psychotherapy (STPP) were identified. In SAD symptoms, STPP was superior to waitlist controls (*k* = 6) with a large pooled effect (*g* = − 0.97, 95% CI: -1.27 to -0.12). Compared with active treatment conditions (*k* = 9), no significant differences were observed (*g* = 0.01, 95% CI: − 0.14 to 0.15). Study quality significantly moderated effects in comparisons with passive controls only, with lower-quality studies reporting larger effects (β = 0.02). No differences in dropout rates were observed. Available follow-up data indicated that gains in social anxiety symptoms were maintained, with some evidence of continued improvement over time.

**Conclusion:**

These findings suggest that STPP may be an effective and acceptable treatment for SAD. Future studies should include active treatment comparisons, assess long-term outcomes, and examine mechanisms of change.

**Trial registration:**

PROSPERO database with ID CRD420250656024.

**Supplementary Information:**

The online version contains supplementary material available at 10.1186/s40359-026-04306-x.

## Background

Social anxiety disorder (SAD), or social phobia, is a highly debilitating mental disorder characterized by an excessive fear of social situations in which the individual may be subject to scrutiny or negative evaluation of others [1]. Prevalence estimates of SAD vary considerably across regions and income levels, ranging from 5.5% in high-income countries to 2.9% in middle-income and 1.6% in the lowest-income countries, reflecting a global lifetime prevalence of approximately 4.0% [2].However, given that avoidance behavior is common among individuals with SAD – including the avoidance of healthcare services [3] – actual prevalence rates may be underestimated. SAD typically has an early onset, with ages ranging from 10.6 to 13 years [4]. Yet symptoms frequently go unrecognized or are misattributed, delaying diagnosis and treatment [5]. On average, individuals struggle with symptoms for several years before seeking and receiving treatment [6]. Without treatment the condition may persist over time, severely impairing daily functioning [7] and reducing quality of life [8].

Individuals with SAD tend to avoid social and performance-related situations, such as those encountered in educational or occupational settings [9]. This pattern of avoidance can result in social isolation, as well as socioeconomic disadvantage (e.g. lower income), which further complicates their already vulnerable position [10]. On a societal level, SAD not only contributes to direct healthcare costs, but also to indirect costs associated with reduced productivity, sick leave, and long-term work disability [11]. The significant impact of SAD on individuals’ daily functioning and quality of life, as well as the high societal costs, underscore importance of effective, tolerable, and accessible treatments. A range of treatment options is currently available for individuals with social anxiety disorder, including well-established interventions such as cognitive behavioral therapy (CBT) [12] and pharmacological treatments [5], as well as less commonly applied approaches like short-term psychodynamic therapy (STPP) [13] and interpersonal therapy (IPT) [14]. Third-wave CBT approaches, including acceptance and commitment therapy (ACT), have also received growing attention in the treatment of SAD in recent years [15]. This range of interventions is based on different theoretical foundations and is assumed to engage partly distinct mechanisms of change, although overlap may exist across approaches [16, 17]. Treatments can be delivered in various formats, such as individual or group-based therapy, as well as face-to-face or (guided) internet-delivered interventions.

CBT is generally regarded as the first-line psychological treatment for SAD. CBT has shown strong efficacy across anxiety disorders, with moderate to large effect sizes compared to pill placebo and waitlist controls [18]. A meta-analysis by Loerinc et al. [19] demonstrated average response rates across anxiety disorders of approximately 49.5% at post-treatment and 53.6% at follow-up. For SAD specifically, response rates were 45.3% at treatment termination and 55.9% at follow-up, suggesting that treatment gains are maintained over time, and generally slightly increased. However, treatment adherence remains an issue: dropout rates for CBT in anxiety disorders have been estimated at 19.6% [20].

Several pharmacological treatment alternatives exist for SAD [21]. Selective serotonin reuptake inhibitors (SSRIs) are generally recommended as pharmacological treatment [5] as they tend to cause fewer side effects and also affect co-occurring depressive symptoms [22]. Still, adverse side-effects are frequently reported and can contribute to non-adherence [5, 23]. Moreover, despite inconclusive evidence, concerns exist about increased suicidality with the use of SSRIs in adolescents and young adults [24–26].

Despite the demonstrated efficacy for CBT and pharmacotherapy [5, 19], a considerable proportion of patients does not experience sufficient improvement, indicating that additional treatment options may be warranted [27]. Psychotherapies grounded in other theoretical frameworks – such as psychodynamic therapy (PDT) – may offer viable alternatives. However, it should be noted that the empirical literature on sequential psychotherapy trials remain limited [28]. That said, a first step towards conducting trials to answer questions regarding what to offer patients who do not respond to a first-line intervention, is the establishment of efficacious treatment alternatives. Moreover, although pharmacological treatments can reduce symptoms, they do not directly address the underlying psychological mechanisms maintaining the disorder, and relapse is common after discontinuation [29]. Beyond rates of non-response, the rationale for researching and developing treatment alternatives is grounded in the ethical principles of patient choice and shared decision-making, which are central to evidence-based practice in psychology [30]. For instance, many patients report a preference for psychotherapy over medication [31]. Research also suggests that patients vary regarding what type of psychotherapy they prefer [32–34]. While CBT and pharmacological treatments are well established for social anxiety disorder, the evidence base for PDT is less developed [35, 36]. PDT seeks to address the underlying psychological mechanisms of the disorder by focusing on unconscious processes, early relational experiences, and internal conflicts [37]. In the treatment of anxiety disorders, moderate to large effect sizes favoring PDT over passive control conditions have been reported [37, 38]. When compared to other treatments such as CBT and pharmacotherapy, PDT appears to be equally effective, with no significant differences in treatment outcomes [38].

Research specifically targeting the efficacy of PDT in SAD, however, remains limited. Two recent meta-analyses have attempted to synthesize the available evidence, but both have notable shortcomings. Zhang et al. [39] reported a standardized mean difference (SMD) of − 0.77 favouring PDT over inactive controls, while no differences were found between PDT and CBT, suggesting that PDT can be a viable alternative treatment. However, their meta-analysis faced extensive criticism for serious methodological flaws, with calls for its retraction [40, 41]. A more recent network meta-analysis by Sun et al. [7] compared a wide range of psychotherapies for SAD but did not include all available randomized controlled trials (RCTs) on PDT, potentially leading to an underestimation of its efficacy. The current meta-analysis aims to address these limitations by offering a more focused and comprehensive evaluation. By including all published RCTs investigating psychodynamic therapy for SAD in adults only, it aims to provide a targeted synthesis of the currently available evidence.

Comorbid depression is frequently observed among individuals with SAD, with prevalence rates estimated between 35% and 70% [42]. The co-occurrence of depression in individuals with SAD has been associated with more severe social anxiety symptoms [43] and greater impairment in daily functioning [44]. Although SAD and depression are distinct disorders, they share overlapping features such as negative self-perception and social withdrawal [45]. While the underlying mechanisms may differ, these symptoms may interact and reinforce one another, potentially exacerbating overall symptom severity [42].

When evaluating the efficacy of PDT for SAD, it is important to consider not only symptom reduction but also broader indicators such as dropout rates, long-term effects, as well as study quality and risk of bias. Dropout rates provide important information about the tolerability of a treatment [46], especially when comparing psychotherapies with different theoretical orientations. Follow-up outcomes, though less frequently reported, are essential for assessing the durability of treatment effects and informing clinical decision-making [4]. Furthermore, study quality has been found to influence effect sizes with low-quality studies often reporting inflated effect estimates alongside larger variability [47]. However, the direction of the impact of potential bias introduced by methodological limitations is often unclear, as it may either inflate or underestimate treatment efficacy [48]. Importantly, this review focuses exclusively on psychodynamic therapy for individuals with SAD, allowing for a more detailed and disorder-specific synthesis than broader meta-analyses that combine multiple diagnoses to achieve statistical power. Given the importance of the above-mentioned factors, they are all included in the current synthesis.

This meta-analysis aims to provide a comprehensive and rigorous evaluation of the efficacy of PDT in the treatment of SAD. The efficacy of PDT will be assessed across symptom domains, alongside dropout rates as an indicator of treatment acceptability. In addition, it will explore follow-up outcomes, as the long-term effects of PDT for SAD remain largely unexplored. If efficacy is demonstrated, PDT may represent a valuable addition to the treatment landscape for SAD. Beyond improving individual well-being and quality of life, effective treatments for SAD may also support reductions in healthcare costs and work-related impairment at the societal level. The following research questions will be addressed: What is the efficacy of psychodynamic therapy in SAD, compared to passive control conditions (i.e. waitlist) and active treatment conditions (i.e. psychotherapy, pharmacotherapy)? Furthermore, the following sub questions will be addressed:

- What is the effect of PDT on the severity of social anxiety symptoms, compared to passive control conditions and active treatment conditions?
- What is the effect of PDT on comorbid depressive symptoms, compared to passive control conditions and active treatment conditions?
- Does study quality moderate the effect sizes of PDT?
- Does dropout rate differ between PDT and other treatment conditions?
- Are the effects of PDT on SAD symptoms maintained in the long-term? (Exploratory)

## Methods

This meta-analysis followed the PRISMA guidelines [49] and was preregistered in the PROSPERO database with ID CRD420250656024. The study protocol was followed as preregistered, with one deviation: the outcome related to quality of life was excluded from the final meta-analysis due to limited data availability.

### Search strategy and eligibility criteria

A comprehensive search strategy was used to identify relevant studies, including both published papers and unpublished manuscripts. Searches were conducted in the following academic databases via EBSCOhost: AMED, APA PsycInfo, APA PsycArticles, ERIC, and Medline. The study selection followed a stepwise process. First, duplicate records were removed. Next, titles and abstracts were screened for relevance. Potentially eligible studies then underwent full-text screening. In addition, a comprehensive list of randomized controlled trials on psychodynamic therapy [50] was consulted, and the reference lists of relevant publications were manually screened for additional eligible studies. Aiming to minimize publication bias, grey literature was searched via ProQuest Dissertations and Google Scholar. Search strings were developed according to the PICO framework as described by Cuijpers [51] and can be found in Appendix A. The ProQuest search followed the same strategy as with the EBSCOhost databases, with additional relevance filters applied (e.g., language, full-text, source type). An adapted search string was used for Google Scholar.

The screening and selection process was conducted by the first author (M.N.) and discussed with (J.M.) to ensure accuracy. Randomized controlled trials conducted between 1980 and the present, published in full-text English, were included if they met the following criteria: psychodynamic psychotherapy delivered individually or in groups, via face-to-face or guided internet-based formats; targeting adults (18+), with social anxiety disorder/social phobia as primary diagnosis, diagnosed according to DSM-III or later, ICD-criteria, or established cut-offs on validated outcome measures; compared to either an active control condition (e.g. other forms of psychotherapy, psychopharmaceutical treatment, pill placebo, care as usual) or passive control condition (e.g., waitlist); and with outcomes including social anxiety symptoms measured on validated scale (e.g., Liebowitz Social Anxiety Scale [LSAS] [52], Social Phobia and Anxiety Inventory [SPAI] [53]) as well as measures of depression (e.g., Beck Depression Inventory [BDI] [54], or the Patient Health Questionnaire-9 [PHQ-9] [55]) and quality of life (e.g., World Health Organization Quality of Life Questionnaire [WHOQOL] [56]). Studies were excluded if they included participants under the age of 18; if SAD was not the primary diagnosis; the PDT intervention was provided in an unguided internet-based format; if no comparison group was included, or no random assignment of participants was used; or if outcome data needed for the primary analyses were not provided.

### Data extraction

Relevant data for study characteristics, moderator variables and calculation of effect sizes were extracted and recorded in an Excel spreadsheet (Version 16.95.1) by the first author. If essential data were missing, study authors were contacted to request additional information. When available, linear mixed model (LMM) estimates were used in preference to observed data, as they account for missing data and repeated measures. To ensure the integrity of the data extraction process, a second researcher (K. L.) independently coded all included studies to verify the correctness of the initial extraction.

#### Study characteristics

The following study characteristics were extracted: author(s), year of publication, sample size, sample characteristics (average age, gender distribution, primary diagnosis and comorbidities), details of the intervention (e.g. treatment format [individual vs. group], treatment duration [number of sessions and calendar weeks] and delivery method [face-to-face or guided internet-based]), type of control condition (active vs. passive, including the nature of any active comparator intervention), and dropout rates.

#### Outcome data

For the primary outcome – social anxiety symptoms – means and standard deviations from validated measures were extracted from baseline, treatment termination and (where available) follow-up. When studies reported multiple measures of social anxiety, the measure most commonly used across studies or most clearly aligned with the primary outcome was selected to enhance comparability. For the secondary outcome measure – depression symptom severity – a similar procedure was followed, extracting data from baseline, treatment termination and follow-up. Dropout was operationalized as premature treatment discontinuation as reported by the original trial authors. In most studies, this referred to participants who did not complete the treatment protocol after being assigned to a treatment condition. As reporting practices varied across studies in the level of detail provided regarding reasons for termination (e.g., voluntary vs. therapist-initiated discontinuation or practical reasons), dropout in this meta-analysis should be interpreted as a pragmatic indicator of treatment acceptability rather than a precise reflection of specific discontinuation processes.

### Quality assessment

The quality of all included studies was assessed using the Randomized Controlled Trial Psychotherapy Quality Rating Scale (RCT-PQRS) [57]. The PQRS is a 25-item measure that assesses the study quality across five subscales: *description of subjects* (9 items), *outcome measures* (5 items), *data analysis* (5 items), *treatment assignment* (3 items) and *overall quality of study* (3 items). The scale has demonstrated good psychometric properties in its validation study, with a Cronbach’s α of 0.87 and inter-rater reliability (ICC) values of 0.76 for the total score and 0.79 for the omnibus rating, supporting its reliability and validity in evaluating psychotherapy trials. Two assessors (M.N. and J.M.) independently rated the included studies, by scoring each item between 0 (inadequate approach and/or poor documentation) and 2 (adequate approach and full documentation), resulting in a total score (item 1–24). In line with the original validation study, a total score of 24 or higher was considered indicative of adequate methodological quality [57]. An additional omnibus score (item 25) rated the overall quality of the study from 1 (exceptionally poor) to 7 (exceptionally good).

To assess inter-rater reliability, the intraclass correlation coefficient (ICC) was computed using a two-way random-effects model with absolute agreement. As the mean score across both raters was used in subsequent analyses, ICC(2,2) was reported. Based on the guidelines by Koo and Li [58], values above 0.75 are considered good and values above 0.90 are considered excellent. Inter-rater reliability for the total PQRS score and omnibus score was excellent, with ICC(2,2) = 0.997 (95% CI: 0.989 to 0.999), and ICC(2,2) = 0.991 (95% CI: 0.968 to 0.998), respectively.

In addition to the PQRS, study quality was also assessed using four criteria of the Cochrane Risk of Bias 2 tool (RoB 2) [59]: *randomization process*, *allocation concealment*, *blinding of outcome measures* and *handling of incomplete outcome data*. Each study was independently rated by both assessors for each criterion as having low, unclear, or high risk of bias, following the RoB 2 manual and the adaptations for psychotherapy trials provided by Metapsy [60]. Discrepancies in ratings were discussed until consensus was reached. Although the option to involve a third assessor was available, full agreement was achieved on all ratings. Because the direction of potential bias is often unclear – meaning it may inflate or deflate effect sizes [48] – the RoB ratings were not used in any statistical analyses but served to inform the interpretation of the findings.

### Statistical analysis

All statistical analyses were conducted in SPSS (version 29.0.0.0). For all analyses a p-value of < 0.05 indicated significance, unless otherwise specified.

#### Calculation of effect sizes

Effect sizes were calculated as Hedges’ *g*, which adjusts Cohen’s *d* for small sample bias [61], with values of 0.2, 0.5, and 0.8 to be interpreted as small, medium, and large effects, respectively [62]. For the main analysis, between-group Hedges’ *g* was computed for post-treatment social anxiety. For analyses with secondary outcomes, Hedges’ *g* values were calculated for post-treatment depression and log odds ratios (log ORs) for dropout rates. To aid interpretation of results, log ORs can be approximately converted to Cohen’s *d* [63], with log ORs of 0.36, 0.91, and 1.45 corresponding to *d* ≈ 0.2, *d* ≈ 0.5, and *d* ≈ 0.8, respectively. 

For two studies [64, 65], subgroup scores were aggregated. In the study by Rahmani et al. [65] only subscale means for the LSAS-SR (Fear and Avoidance) were reported, without a total score, and the psychodynamic intervention was delivered to two separate subgroups. As no significant differences between the intervention subgroups were observed, and total LSAS-SR scores were more suitable for the purpose of this meta-analysis, pooled means and standard deviations were calculated to combine the two groups. Due to concerns about study quality and the large observed effect sizes, a conservative approach was taken by assuming a correlation of *r* = 1.0 between subscales [63] on LSAS-SR when estimating total SAD scores. Furthermore, to increase power and maintain consistency across studies, the two active comparator conditions from Alström et al. [64] – prolonged exposure in vivo (PE) and relaxation training (R) – were combined into a single active control group. Although the PE group showed slightly greater symptom reduction, the sample size was small (*n* = 7). Furthermore, the PE group exhibited lower baseline SAD severity, and less comorbidity compared to the psychodynamic therapy group (*n* = 16), which may have confounded direct comparisons. Combining both groups provided a more balanced and representative control condition for inclusion in the current meta-analysis. The combined standard deviation was calculated using the formula for pooled standard deviations for independent subgroups, as recommended by Borenstein et al. [63].

#### Meta-analysis and heterogeneity assessment

Two separate random-effects meta-analyses were conducted to estimate the treatment effect of psychodynamic therapy on social anxiety disorder (SAD): one comparing PDT to passive control conditions (i.e. waitlist) and one comparing PDT to active comparators. Between-study heterogeneity was assessed using multiple indicators. First, Cochran’s Q-test [66] was used to examine whether observed heterogeneity was greater than expected by sampling error alone. Following common practice in meta-analysis, a significance threshold of *p* < .10 was applied [67]. Second, the *I²* statistic quantified the proportion of total variance due to heterogeneity rather than chance, with values of ≤ 25%, 25–50%, and ≥ 75% interpreted as low, moderate, and high heterogeneity, respectively [68]. Third, τ² (tau-squared) was calculated as an estimate of between-study variance, using the Sidik-Jonkman [69] method, which has shown to perform more reliably in studies with considerable heterogeneity. A Knapp-Hartung [70] adjustment was applied to obtain more accurate confidence intervals. This combination of the Sidik–Jonkman estimator with Knapp–Hartung adjusted inference is recommended for meta-analyses with few studies [71].

#### Moderator analysis

To check for potential baseline differences across studies, standardized mean scores for social anxiety and depression at pre-treatment were examined. For social anxiety, some between-study variation in baseline severity was observed, likely reflecting differences in outcome measures. No notable differences were found in baseline depression levels across studies. Although it is theoretically plausible that baseline symptom severity could influence treatment outcomes, these variables were not included as moderators, as meta-analysis on the study-level is not an appropriate method to answer this question.

To evaluate whether study quality moderated treatment effects, two separate univariate meta-regressions were performed for the active and passive comparison groups. Only the total quality score, not the omnibus score, was used in the meta-regression, as is conventional in other meta-analytic reviews [37, 72]. Since the total and omnibus scores were highly correlated (*r* = .98), no meaningful differences in findings were expected. In addition to the analysis using total PQRS score as a continuous moderator, a post-hoc analysis was conducted treating study quality as a dichotomous variable (adequate [total score ≥ 24] vs. lower quality) to examine whether the findings were sensitive to an alternative operationalization of study quality.

#### Sensitivity analysis and publication bias

In line with the preregistration, outcomes for individual versus group-based PDT were compared, as group-based interventions have been found to yield lower effect sizes in psychotherapy research [73]. Additionally, internet-delivered therapy was examined as an extra subgroup, as this therapy format was also represented in the dataset. The exploratory subgroup analyses were performed separately for passive and active comparators, allowing for a more nuanced interpretation of the found effect sizes. Leave-one-out analyses were not performed, as statistical heterogeneity was low in the primary outcome meta-analyses. Given the conceptual heterogeneity among active comparator interventions, an additional post-hoc subgroup analysis was conducted including only studies comparing STPP with CBT. Due to the limited number of studies involving pharmacotherapeutic treatments, no post-hoc analyses were performed for this subgroup.

Publication bias was examined in three steps. First, funnel plots were visually inspected for the primary outcome analyses to assess asymmetry, which may indicate publication bias. Second, Egger’s regression test [74] was applied, with statistically significant results suggesting funnel plot asymmetry. Third, the trim-and-fill method [75] was used to estimate the number of potentially missing studies on the asymmetric side of the funnel plot and to adjust the effect size by imputing these studies to restore symmetry.

#### Secondary outcomes

To assess effects on depression severity at treatment termination, random-effects meta-analyses were conducted comparing PDT to passive and active control conditions. Meta-regression was not performed due to the limited number of studies reporting depression outcomes (*k* = 8). Differences in dropout rates between PDT and active comparison groups were assessed by pooling the log odds ratios of treatment dropout using a random-effects model.

### Exploring long-term effects

To explore the potential long-term effects of psychodynamic therapy, both between-group and within-group meta-analyses were performed using the latest available follow-up timepoint. Effect sizes for the follow-up analyses were calculated from observed data, as no LMM estimates were reported for these assessments. Random-effects models were conducted using the Hartung-Knapp-Sidik-Jonkman method [71]. To contextualize the robustness of the follow-up findings, cumulative dropout rates across all follow-up assessments were summarized, as most studies relied on intention-to-treat analyses and missing data may affect the reliability of long-term outcome estimates.

## Results

### Study selection

**Fig. 1:**
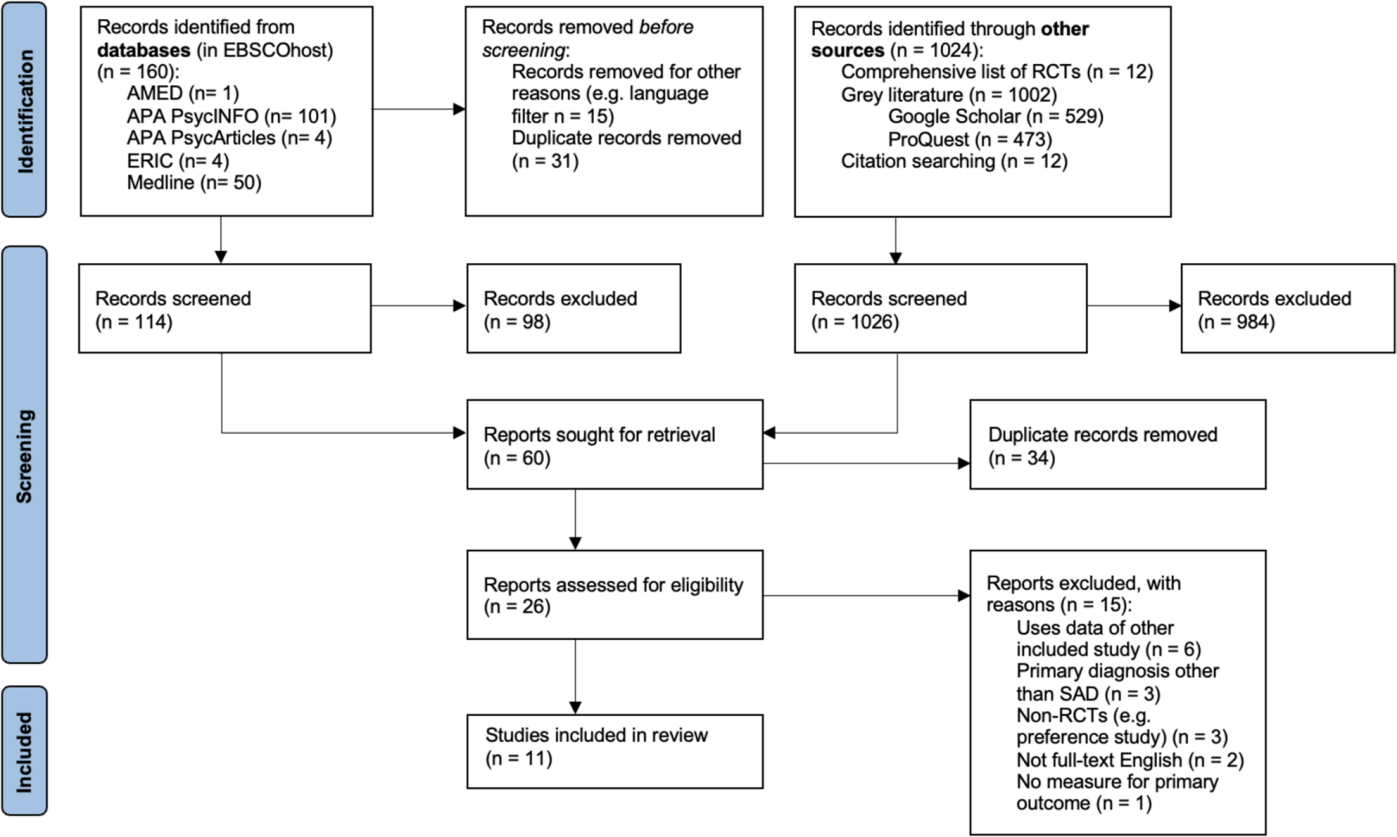
PRISMA flow chart describing the selection and inclusion process

Database searches were performed on 2 March 2025 accessing EBSCOhost and ProQuest through Uppsala University Library. Following removal of duplicates and screening of abstracts, 26 reports were assessed for eligibility. Of these, 11 studies met all inclusion criteria and were included in the final review. One of these studies was reported in two articles [76, 77], the first describing the initial treatment phase and the second presenting long-term outcomes at follow-up. During full-text screening, two studies required careful evaluation due to initial uncertainty regarding eligibility. First, Rahmani et al. [78] was excluded because it concerned a sub-study based on the same sample as another included study [65]. Second, Wiltink et al. [79] was excluded because patients were not randomly assigned to treatment conditions; instead, only therapists were randomized while patients could choose among therapists, thus violating our random assignment criteria. Reasons for exclusion of the remaining 11 studies are detailed in the PRISMA flow chart (Fig. 1). 

### Characteristics of the included studies

Although the review aimed to include all forms of PDT for SAD, all included studies evaluated short-term psychodynamic psychotherapy, meaning that the findings may not generalize to longer-term psychodynamic treatments. Results regarding the PDT interventions are therefore referred to as STPP from this point forward. The included studies – as presented in Table 1 – were published between 1984 and 2024 and were conducted in Sweden (*k* = 3), Brazil (*k* = 3), Iran (*k* = 2), and one study each in the Netherlands, Germany, and the United States. Although most psychodynamic approaches included Malan’s [80] two-triangle model as a theoretical foundation – aiming to identify patterns of defense, anxiety, and underlying emotional needs – distinct therapeutic focuses were applied across the studies. Several studies [65, 81–84] applied a form of Experiential Dynamic Therapy (EDT) [85]. EDT is a short-term psychodynamic approach that emphasizes the processing of core emotional experiences by helping patients overcome defenses and regulate anxiety, using the here-and-now relationship between therapist and patient to facilitate affective breakthroughs [86]. In contrast, two studies [64, 87] implemented supportive dynamic therapy, which focused on strengthening patients’ self-esteem and coping abilities through empathic listening, affirmation and encouragement rather than on deep exploration of unconscious conflicts. Finally, Leichsenring et al. [76, 77] applied a manual-guided psychodynamic treatment developed for SAD [13], based on Luborsky’s [88] Supportive-Expressive Therapy.

**Table 1:**
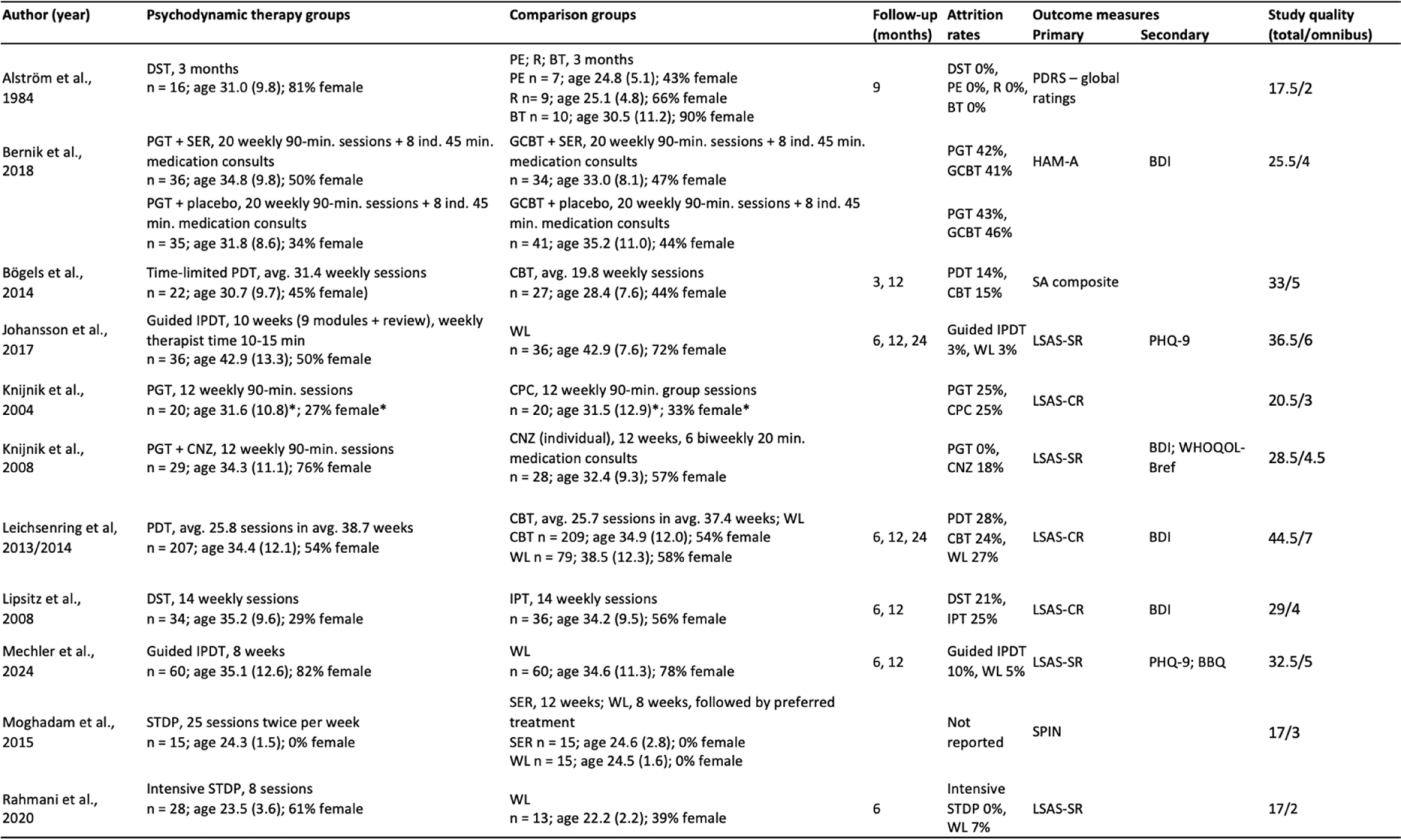
Study characteristics [64, 65, 76, 77, 81–84, 87, 89–91]

*BDI* Beck Depression Inventory, *BBQ* Brunnsviken Brief Quality of Life Scale, *BT* Basal Therapy, *CBT* Cognitive Behavior Therapy, *CNZ* Clonazepam, *CPC* Credible Placebo Control, *DST* Dynamic Supportive Therapy, *GCBT* Group Cognitive Behavior Therapy, *HAM-A* Hamilton Anxiety Rating Scale, *IPDT* Internet-based Psychodynamic Therapy, *IPT* Interpersonal Therapy, *LSAS-SR/CR* Liebowitz Social Anxiety Scale - Self-report/Clinician-rated, *PDRS* Phobic Disorders Rating Scale, *PDT* Psychodynamic Therapy, *PE* Prolonged Exposure in vivo, *PGT* Psychodynamic Group Therapy, *PHQ-9* Patient Health Questionnaire 9, *R* Relaxation, *SA composite* Social Anxiety composite, *SER* Sertraline, *SPIN* Social Phobia Inventory, *STDP* Short-Term Dynamic Psychotherapy, *WL* Waitlist, *WHOQOL-Bref* World Health Organisation Quality of Life Brief Version * Based on treatment completers (n = 15 per group)

Regarding comparison groups, nine studies included active controls and six included passive controls. Since all the treatment arms in Alström et al. [64] received basal therapy alongside a specific intervention, the group receiving only basal therapy was considered a passive control. Active control groups consisted of one of the following treatments: CBT, IPT, credible placebo control (i.e. educational-supportive group therapy without structured therapeutic elements) or a form of pharmacotherapy. Treatments were mainly delivered in face-to-face format, except for Johansson et al. [82] and Mechler et al. [83], where guided internet-based therapy was used. Most therapies were conducted individually; however, Bernik et al. [89] and Knijnik et al. [90, 91] delivered STPP in a group setting.

The duration of treatments ranged from 8 to 31 (often weekly) sessions in STPP conditions and 12 to 20 weekly sessions in psychotherapy control groups. Pharmacological control groups followed the treatment duration of the STPP intervention. Treatments were delivered in different study contexts, ranging from university-based trials to clinical RCTs. Recruitment strategies varied accordingly, resulting in heterogeneous samples across studies. Clinical samples and individuals actively seeking treatment typically exhibit more severe social anxiety symptoms and higher rates of comorbidity compared to those recruited through more passive methods, such as newspaper advertisements, on-campus outreach, or referrals via friends and family [92].

Several studies reported the presence of comorbidities such as other anxiety disorders, depression and personality disorders. Six studies included depression symptom measurements. To assess social anxiety symptoms, the majority of studies (64%) used a version of the LSAS [93]. However, two studies used instruments developed and validated specifically for the trial [64, 81, 94]. One study [89] used a general anxiety measure – the Hamilton Anxiety Rating Scale [95]. Finally, only two studies [83, 91] included assessments of quality of life, limiting the possibility to draw firm conclusions regarding treatment effects on this outcome. Follow-up assessments were most often conducted at 6 and 12 months, with Johansson et al. [82] and Leichsenring et al. [77] also presenting data from 2-years after treatment termination. Considerable variation in attrition rates was observed across studies, ranging from 0% to 42% in both trial arms during the treatment phase, with an average of 16.9% in STPP conditions. At follow-up assessments in the STPP groups, average attrition rates were 18.4% at 3–6 months, 27.1% at 9–12 months, and 51.7% at 2 years after treatment termination.

### Effects of STPP social anxiety

#### Passive control groups

**Fig. 2:**
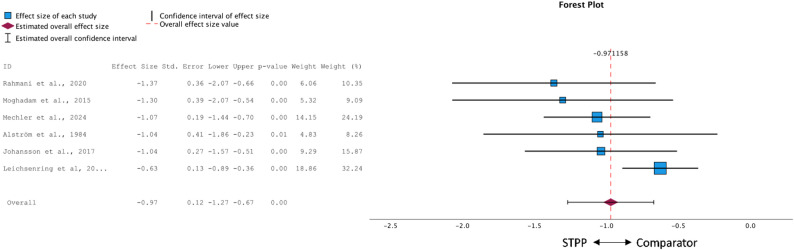
Forest plot of between-group effect sizes of STPP compared to waitlist

When comparing to passive control groups (*k* = 6), STPP interventions were superior to waitlist conditions, yielding a large effect at treatment termination (*g* = − 0.97 [95% CI: -1.27 to 0.12], *p* < .001). Although the Q-test turned out non-significant (*Q* = 7.99, *p* = .16), an *I²* of 36% indicated moderate heterogeneity, consistent with the wider spread of effect size estimates shown in the forest plot (Fig. 2). Slight asymmetry of the funnel plot was observed and Egger’s regression test approached but did not reach significance (*p* = .075). The trim-and-fill procedure imputed two studies on the right side of the funnel (see Appendix B, Fig. 1), resulting in an adjusted effect of *g* = − 0.89 (95% CI: -1.18 to − 0.60, *p* < .001).

#### Active comparison groups

**Fig. 3:**
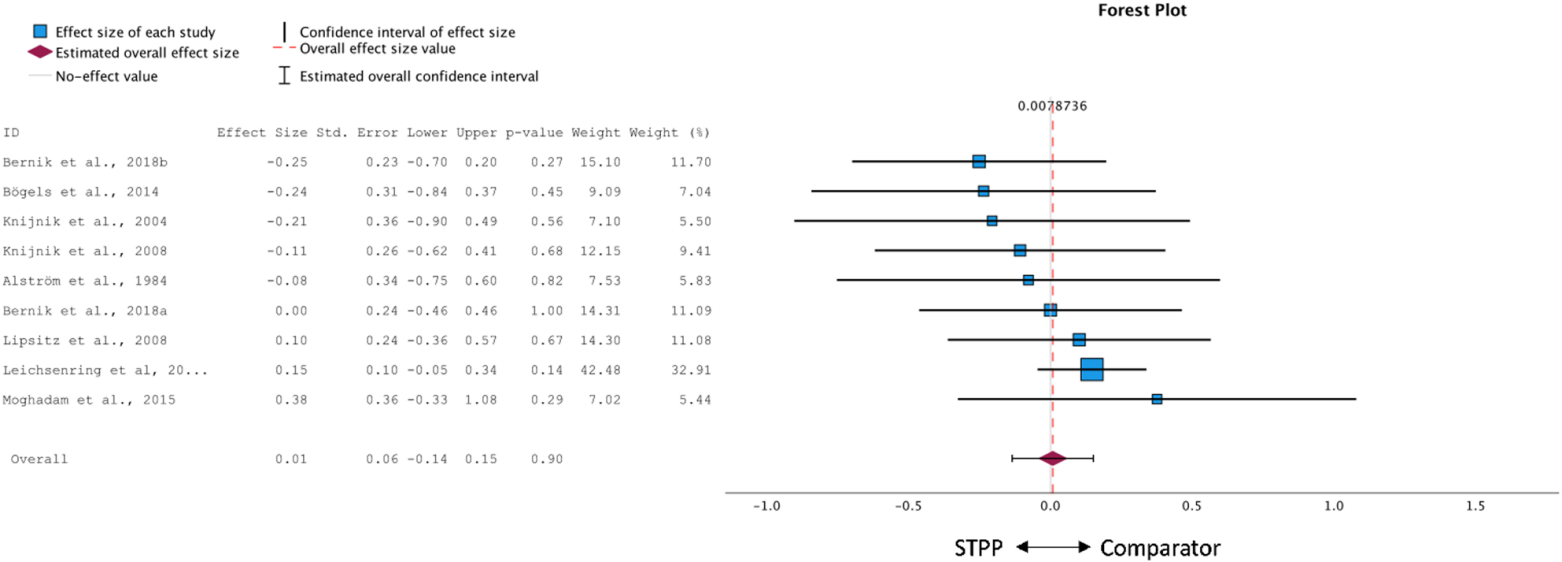
Forest plot of between-group effect sizes of STPP compared to active comparisons

The meta-analysis comparing STPP to active treatment conditions (*k* = 9; see Fig. 3) showed a pooled effect size of *g* = 0.01 (95% CI: − 0.14 to 0.15), suggesting no significant differences in treatment outcomes (*p* = .902). The Q-test was non-significant (*Q* = 5.48, *p* = .71), and both *τ²* = 0.01 and an *I²* of 21% suggested low heterogeneity across studies. Visual inspection of the funnel plot revealed some asymmetry. Egger’s regression test, however, was non-significant (*p* = .261). The trim-and-fill procedure imputed three studies on the right side of the funnel (see Appendix B, Fig. 2). After imputation the pooled effect remained non-significant, *g* = 0.08 (95% CI: − 0.07 to 0.24, *p* = .264).

The post-hoc sensitivity analysis comparing STPP with CBT (*k* = 5) yielded a pooled effect size of *g* = 0.00 (95% CI: − 0.23 to 0.23), which was not significant (*p* = .974). Heterogeneity within this subgroup was low (*Q* = 3.74, *p* = .443; *I²* = 19.5%), suggesting only minor statistical variation across the included studies. Results of this analysis are summarized in a forest plot provided in the supplementary material (Appendix C).

### Exploring variation in outcomes across therapy formats

Exploratory subgroup analyses examined whether treatment format was associated with differences in effect sizes. For active comparison groups, two therapy formats were represented: individual (*g* = 0.09, 95% CI: − 0.12 to 0.31, *p* = .300) and group-based STPP (*g* = − 0.14, 95% CI: − 0.32 to 0.06, *p* = .109). Although both the formats show small effects in opposite directions, the between-subgroup Q-test indicated no significant differences (*Q* = 1.70, *p* = .19).

For passive control comparisons, individual STPP showed a pooled effect of *g* = − 0.96 (95% CI: -1.56 to − 0.36, *p* = .014), with effect sizes ranging from small to large across studies. Internet-based STPP demonstrated a more homogeneous pattern, resulting in a large pooled effect of *g* = -1.06 (95% CI: -1.21 to − 0.90, *p* = .008). However, treatment format did not account for significant differences in effect sizes in comparisons with waitlist controls (*Q* = 0.14, *p* = .71).

### Secondary outcomes

#### Depression

Eight studies included a measure of depression as secondary outcome. Meta-analyses revealed similar patterns as observed for the primary outcome, aligning both in direction and magnitude of the effects. Compared to passive controls (*k* = 3), STPP showed a significant moderate effect in reducing depressive symptoms at treatment termination (*g* = − 0.71, 95% CI: -1.20 to − 0.21, *p* = .025). An *I²* of 35% indicated moderate heterogeneity, while the Q-test suggested no significant between-study differences (*Q* = 2.55, *p* = .28). The analysis comparing STPP with active treatment groups (*k* = 5) showed a pooled effect size of *g* = 0.08 (95% CI: − 0.17 to 0.34), indicating no significant differences between both conditions (*p* = .419). Heterogeneity was low to moderate (*τ²* = 0.02; *I²* = 37%), with a non-significant Q-test (*Q* = 3.97, *p* = .41) suggesting that effect sizes were relatively consistent across studies. Forest plots for depression outcomes are presented in Appendix D.

#### Dropout rates

**Fig. 4:**
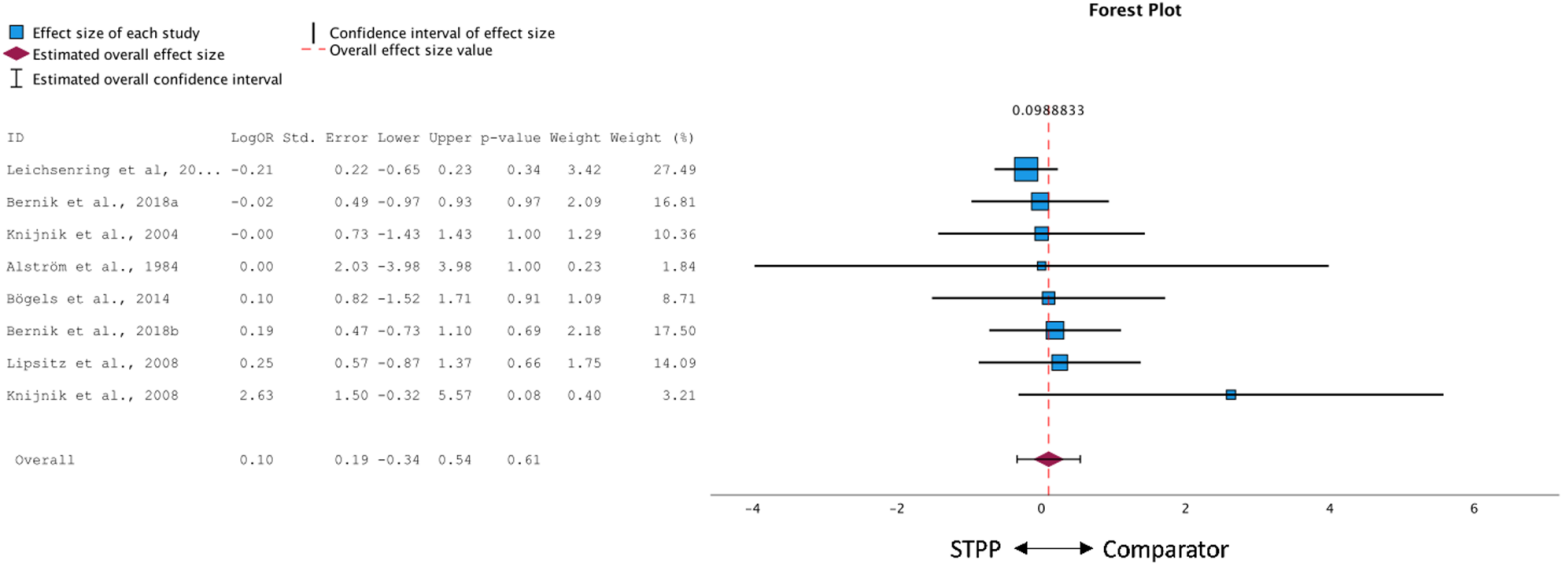
Forest plot of dropout rates in STPP compared to active comparisons at treatment termination

Across nine studies with active comparator groups, eight provided sufficient attrition data for analysis. One study [84] was excluded due to inconsistent reporting of dropout and completion rates. No significant differences in dropout rates were found between STPP and other interventions (LogOR = 0.10, 95% CI: − 0.34 to 0.54, *p* = .610). The Q-test suggested no significant between-study variability (*Q* = 4.27, *p* = .748), although moderate heterogeneity was indicated by an *I²* of 45%, as visualised in the forest plot (see Fig. 4). A trim-and-fill procedure did not impute additional studies.

### Long-term effects on social anxiety symptoms

Exploratory meta-analyses examined whether treatment effects were maintained at the latest available follow-up assessment. Three studies were included in the between-group analysis, with follow-up periods ranging from 9 months [64] to 24 months [77]. A pooled effect size of *g* = − 0.01 (95% CI: − 0.25 to 0.24) indicated no significant differences between STPP and comparison treatments at follow-up (*p* = .926). Heterogeneity was minimal (*I²* = 7%), and the Q-test was non-significant (*Q* = 0.62, *p* = .73).

The within-group analysis included all studies providing sufficient follow-up data (*k* = 6), with follow-up periods ranging from 6 months in Rahmani et al. [65] to 24 months [82]. The pooled effect size suggested small but non-significant improvements from post-treatment to follow-up (*g* = 0.17, 95% CI: − 0.08 to 0.42, *p* = .146), indicating that treatment effects were maintained over time. The Q-test indicated no significant variability across studies (*Q* = 3.91, *p* = .562), while an *I²* of 34% suggested moderate heterogeneity.

Dropout rates were variable across studies and follow-up timepoints. While several studies maintained relatively low attrition (e.g., 0% in Rahmani et al. [65], at six months), others experienced substantial loss to follow-up. Leichsenring et al. [76, 77] in particular reported high dropout rates, with 48% attrition at six months and up to 81% at the 2-year assessment. Although long-term outcomes were analysed using ITT-methods, such extensive attrition limits the reliability and interpretability of these findings.

Taken together, the exploratory results suggest that there is no difference between STPP and other active treatments in long-term treatment effects. Furthermore, the results suggest that symptom reductions observed at post-treatment were generally maintained over time, with some indications of gradual continued improvement on the long-term. However, these findings should be interpreted cautiously due to the small number of included studies, variation in follow-up durations and the methodological limitations inherent to within-study comparisons.

### Study quality and risk of bias

Based on the individual scores of the two assessors, inter-rater averages of both PQRS total and omnibus scores were computed for each study. Across the included studies the total PQRS scores ranged from 17 to 44.5 (of a possible 48), with a mean of 27.41 (SD = 8.95) indicating that, on average, studies exceeded the cut-off score of 24 for adequate methodological quality. Omnibus ratings ranged from 2 to 7, indicating very poor to exceptionally high study quality. Common methodological limitations included small sample sizes, with eight studies having fewer than 40 participants per arm, thereby limiting statistical power. Furthermore, seven studies failed to report information on relevant comorbidities of participants. The individual ratings of both assessors are presented in Appendix E.

**Fig. 5:**
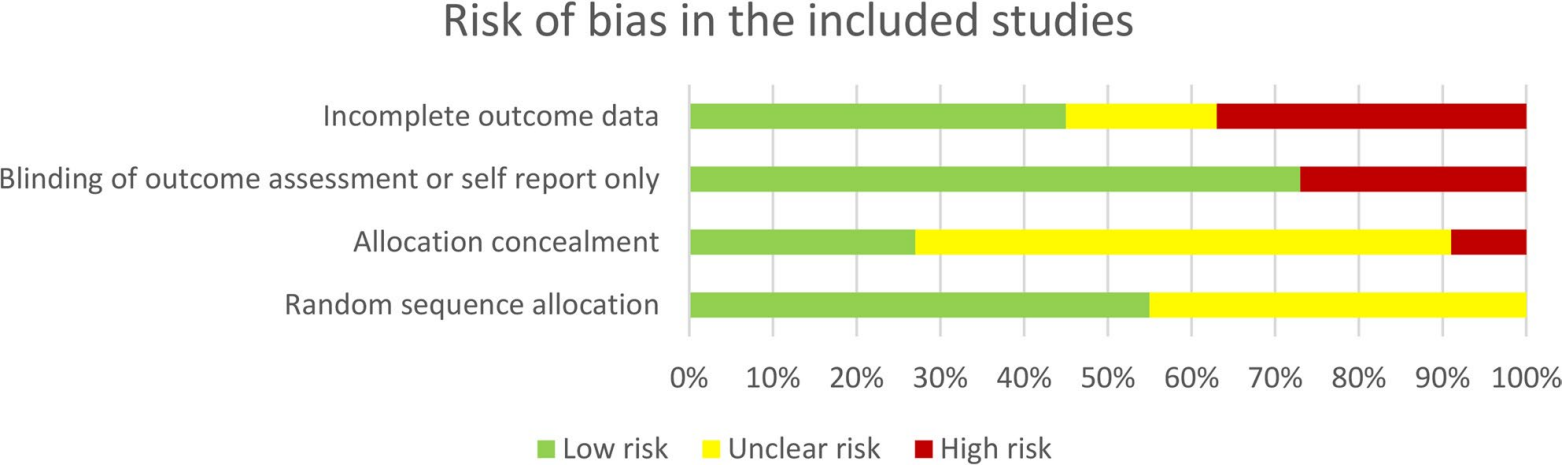
Summary of ratings on the four cochrane risk of bias criteria

Results of the risk of bias assessment using the four Cochrane criteria are summarized in Fig. 5. The highest risk was observed for the criterion concerning the handling of incomplete outcome data (i.e., not using a full intention-to-treat (ITT) analysis), with four studies (37%) rated at high risk [65, 84, 87, 90], and two studies (18%) at unclear risk for reporting partial or adjusted ITT-protocols [64, 76, 77]. Regarding blinding, eight studies met the criterion for blinding of outcome assessment, of which three studies [65, 82, 83] relied solely on self-report measures. According to the RoB guidelines for psychotherapy trials [60] patient-rated measures are considered ‘blinded’, as they appear to produce more conservative results than clinician ratings [96].

Concerning randomization, all studies reported that randomization was conducted. However, almost half of the studies failed to provide sufficient information about the randomization process, and 64% lacked adequate reporting of allocation concealment. As a result, it remains uncertain whether full randomization procedures were properly implemented across all studies. Only two studies [82, 83] showed low risk of bias on all four criteria, while two studies [64, 84] met none of the criteria. A detailed overview of the ratings per criterion and per study is provided in Appendix F.

### Study quality as moderator of effect size

**Fig. 6:**
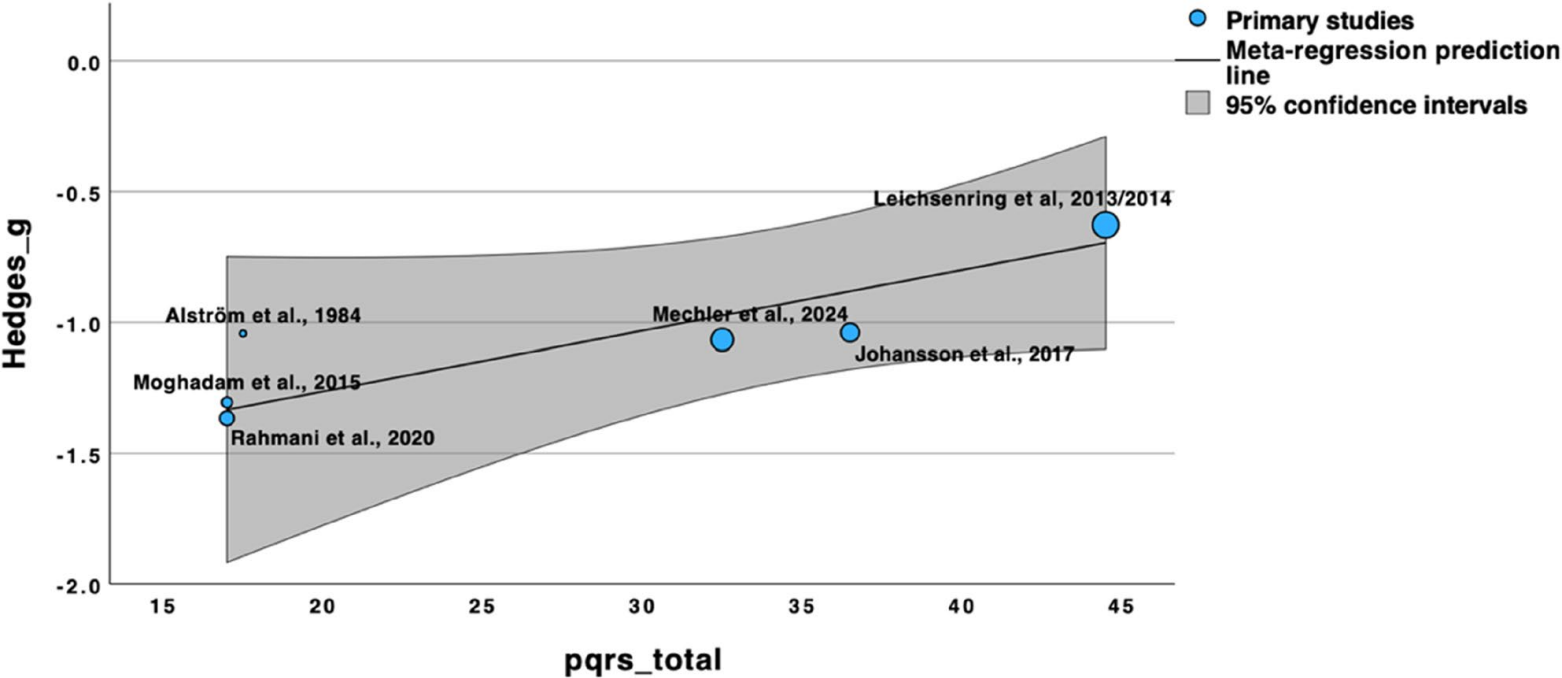
Lower-quality studies show larger effect sizes for STPP versus passive controls

Study quality did not significantly moderate effect sizes for active comparators (*F* (1, 7) = 1.22, *p* = .305), which aligns with the small range of effect sizes observed in this comparison group. For passive control comparisons, however, study quality significantly predicted effect size with *F* (1, 4) = 18.41, *p* = .013. Lower-quality studies reported larger effects (β = 0.02, 95% CI: 0.01 to 0.04), as is illustrated in the bubble plot (Fig. 6). The model explained 72.8% of the between-study variance, leaving only a small proportion of the heterogeneity unexplained (*I²* = 12%). As the analysis only included six studies, the robustness of this moderator analysis is limited. In the post-hoc sensitivity analysis treating PQRS as a dichotomous variable (adequate vs. lower quality), the moderation effect for passive controls was no longer statistically significant (*F* (1, 4) = 3.03, *p* = .157).

## Discussion

The current meta-analysis aimed to explore the potential of PDT as a treatment option for individuals with social anxiety disorders by integrating all available randomized controlled trials on this topic. Interestingly, all included studies applied a form of short-term psychodynamic psychotherapy. Treatment effects of STPP on social anxiety and comorbid depressive symptoms, as well as dropout rates, were compared against passive control and active treatment conditions. Furthermore, exploratory analyses assessed whether improvements were sustained over the longer term. As expected, STPP outperformed waitlist control groups with moderate to large effects, while showing broad equivalence to active comparators. Treatment gains were maintained over time, with indications of gradual further improvement.

The presented results must be interpreted in context. There was considerable variation across studies in sample sizes, comparator types (e.g., CBT, pharmacotherapy, credible placebo group therapy), and methodological quality, as well as a limited number of studies per subgroup. The fact that some studies combined psychotherapy with medication further contributed to the heterogeneity across comparison conditions. For instance, Bernik et al. [89] compared psychodynamic group therapy and group CBT, complemented by either sertraline or placebo medication. Although we analyzed the sertraline and placebo arms separately, the combination may have introduced variance unrelated to the psychotherapy modality itself. Similarly, Knijnik et al. [91] compared group psychotherapy plus clonazepam to individual clonazepam, introducing differences in both treatment modality and format. Knijnik et al. [90], on the other hand, used a credible placebo comparator: a group intervention without structured therapeutic content, presented as psychotherapy. Although we classified this as an active treatment due to participants’ treatment expectations, it reflects the challenges of defining adequate controls. While such cases were few and unlikely to meaningfully bias results, they underscore the heterogeneity of comparator conditions and the need for future trials to use more consistent, clearly defined control groups to facilitate comparison and interpretation.

In an attempt to explore the impact of heterogeneity among active control interventions, we conducted a post-hoc sensitivity analysis including only studies comparing STPP with CBT (*k* = 5), which did not reveal a significant difference. Due to the limited number of studies with pharmacotherapy-only comparators, no such comparison could be performed for this group. Similarly, although the exploratory subgroup analysis on treatment format revealed small differences between group- and individual-based interventions, the within-subgroup differences of STPP compared to active controls were minor and non-significant. Considering these findings in light of the primary aim of this meta-analysis – exploring the potential of PDT as a treatment option for SAD – the results provide support that STPP may be an effective intervention, though firm conclusions regarding comparative efficacy require further research.

The variability in comparator conditions highlights the broader issue of heterogeneity in psychotherapy research, which also applies to differences among STPP interventions themselves. In the current meta-analysis, the included studies applied slightly different psychodynamic protocols, including experiential dynamic therapy [86], dynamic supportive therapy [64, 87], and supportive-expressive therapy [88]. Despite these differences, the interventions share core psychodynamic principles, such as addressing unconscious conflicts, exploring interpersonal patterns and defense mechanisms, and using the therapeutic relationship to facilitate emotional insight [97]. In STPP, these principles are complemented by an active therapeutic stance, the early definition of a treatment focus, and an emphasis on the here-and-now [98]. The shared theoretical foundations across approaches support their aggregation in the present meta-analysis to evaluate their overall efficacy in treating SAD. At the same time, clarifying potential differences between specific STPP protocols remains an important direction for future research.

Despite the variability across studies, the main findings are broadly consistent with earlier meta-analyses on psychodynamic therapy for anxiety disorders [37, 38], where similar patterns of results were observed. Moreover, a recent network meta-analysis of Sun et al. [7] found good efficacy for PDT, identifying it as the most effective psychological treatment alternative to CBT. Although their analysis relied on a different methodological approach – comparing various treatments relative to waitlist rather than direct head-to-head comparisons – its conclusion strengthens confidence in the robustness of our findings. In line with this, Zhang et al. [39] also reported comparable effects in passive and active comparison groups, despite the noted methodological limitations (e.g., inclusion of ineligible studies and exclusion of eligible ones). Compared to CBT, which shows moderate to strong effects in prior meta-analyses (e.g. *g* = 0.98 against waitlist [*k* = 40], and *g* = 0.44 to 0.48 against care-as-usual [*k* = 3] and pill-placebo [*k* = 5]) [18], our analysis found no significant differences between STPP and active control conditions, while yielding effects of almost similar magnitude against waitlist controls. It is important to note, however, that the active control subgroup in Cuijpers et al. was limited and potentially underpowered, similar to our sample, although we had a larger proportion of active comparison conditions (*k* = 9 out of 15 comparisons). Thus, while CBT appears to demonstrate stronger effects against active comparators, both bodies of evidence warrant cautious interpretation. Taken together, the convergence of findings across reviews further supports the potential of STPP as a valuable treatment for social anxiety disorder.

The secondary meta-analysis examining depressive symptoms showed effects broadly similar to those observed for social anxiety. Improvements were generally larger compared to passive controls and did not differ significantly from active treatments. Previous research has also reported that psychodynamic therapy can reduce depressive symptoms [38]. Other literature suggests that psychotherapy not only addresses primary symptoms but also has an impact on comorbid symptoms [99]. However, as the current meta-analysis does not examine the potential moderating role of comorbid depression in SAD outcomes, its impact remains unclear and warrants further investigation [43, 45].

In the present meta-analysis, the average PQRS score across studies was 27.41, exceeding the threshold for adequate quality [57] and similar to the average quality observed in CBT trials (*M* = 25.7) [47]. This finding challenges the common perception that psychodynamic therapy is not supported by methodologically sound research [100]. Still, it is important to recognize that efficacy research on psychodynamic therapy – in general and more specifically in SAD – is at an early stage. While CBT has built a substantial evidence base (e.g., Cuijpers et al. [101] identified 46 CBT comparisons for SAD; Loerinc et al. [19] reviewed 87 studies on anxiety disorders), far fewer trials have investigated PDT for SAD [50]. Nonetheless, the existing PDT studies show, on average, at least adequate methodological quality [102], suggesting that future research in this field can build on a growing and methodologically sound foundation.

The risk of bias assessment revealed a mixed and somewhat more concerning pattern. Only two studies met all four Cochrane criteria, and most showed moderate risk of bias, mainly due to poor reporting of allocation concealment and incomplete outcome data (e.g., lacking or partial ITT analyses). While some of these limitations may reflect underreporting rather than true methodological flaws, they still reduce transparency. Compared to Öst’s [103] meta-analysis on CBT for anxiety disorders, which included a substantial proportion of low-risk studies *(k* = 8), the present sample appears to have a higher proportion of studies with moderate or unclear risk, reflecting the need for improved reporting standards in future trials on psychodynamic therapy. That said, by combining both PQRS and risk of bias evaluations, this meta-analysis offers a nuanced assessment of how methodological factors might have impacted the results. Whereas bias assessments focus on systematic error (e.g., selection, attrition, and reporting bias) [51], the PQRS [57] captures broader quality aspects of the study design including contextual factors such as therapist training and allegiance.

Study quality was a significant moderator when comparing STPP to passive controls but did not significantly moderate effects against active comparators. These results are consistent with findings from Lilliengren et al. [86] and align with Thoma et al. [47], who noted that lower-quality studies tend to produce inflated effect estimates with greater variability. In contrast, Keefe et al. [37] found no significant associations between study quality and treatment effects. Moreover, other authors have highlighted that bias – closely related to study quality – may distort results in either direction [48]. Minor methodological differences across studies, such as how quality was assessed or operationalized in the analyses, further complicate interpretation, underscoring the complexity of evaluating study quality as a moderator of treatment effects.

In the present meta-analysis, study quality was primarily modeled as a continuous variable to retain variability and allow for a more nuanced examination of its association with effect size. When study quality was dichotomized (adequate vs. lower quality) in post-hoc sensitivity analyses, the moderation effect for passive controls was no longer significant. Given the limited number of studies (*k* = 6) dichotomization may have reduced statistical sensitivity by collapsing a wide range of quality scores into a single category (e.g., studies scoring 25 and 44 out of 50 on the PQRS would both be considered “adequate”). Study quality is a multifaceted construct, and although it cannot be assumed to operate as a strictly linear dimension, treating it as continuous better preserves variability and captures subtle patterns in effect sizes. Nevertheless, the sensitivity of the findings to how study quality was operationalized warrants cautious interpretation. The current results suggest a tendency for higher-quality studies to report more moderate effect sizes when STPP was compared to passive controls. However, as the PQRS [57] comprises items reflecting different aspects of methodological quality, it remains unclear which specific elements contributed most to this association. Further psychometric evaluation of the PQRS, as well as meta-analytic studies with a larger evidence base could help clarify the best approach to modeling study quality in moderator analyses.

Moving forward, the present meta-analysis found no significance differences in dropout rates between psychodynamic therapy and comparator conditions, suggesting that STPP may be an acceptable treatment option for individuals with SAD. This finding is consistent with earlier research showing comparable dropout rates across psychotherapeutic approaches. With an average dropout rate of 16.9%, the current meta-analysis shows similar attrition levels to those reported for CBT in anxiety disorders (19.6%) [20] and across various psychotherapies for anxiety disorders (16.2%) [104]. However, this rate should be interpreted cautiously, given methodological limitations such as the small number of included studies and variation in comparator conditions.

Worth mentioning is that the reasons for dropout were not consistently assessed and reported across studies, limiting conclusions about treatment tolerability or barriers to adherence. Future trials would benefit from systematically capturing the reasons for attrition, as this information could offer insights into not only the accessibility and acceptability of treatments, but also their limitations [46]. Treatment discontinuation may be influenced by a range of factors, including treatment factors (e.g., intensity, format, theoretical orientation), patient characteristics, socioeconomic barriers, and individual preferences or assumptions about therapy [105]. Notably, treatment preference itself has been associated with both improved adherence and better outcomes [106]. Overall, while acknowledging the limitations of the current meta-analysis, the present findings may be interpreted as an indication that STPP is, on average, as acceptable to patients as other treatment options for SAD.

As only few studies provided follow-up data for both STPP and comparison groups, both within- and between-study analyses were conducted to explore long-term effects. While no significant differences between STPP and active treatments were found, within-study analyses indicated that symptom reductions achieved at post-treatment were generally maintained or slightly improved over time. Comparable findings have been reported in previous literature. For instance, Kindred et al. [107] found that CBT for SAD demonstrated sustained improvements at follow-up. Similarly, Barber et al. [38] observed no significant differences between PDT and other active treatments at short- or long-term follow-up, further supporting the view that PDT can produce durable effects. In contrast, pharmacological treatments have yielded more variable long-term outcomes [5]. While some benefits may persist, 30–50% of the gained improvement is often lost after discontinuation, suggesting that continued medication is needed to maintain the effect [22]. However, long-term pharmacological treatment is generally discouraged in clinical guidelines for SAD [36], as potential benefits are outweighed by adverse side effects and availability of more effective treatment alternatives [108].

Taken together, these exploratory findings suggest that PDT in general, and STPP specifically, may offer durable benefits for individuals with SAD, potentially comparable to those of other established treatments. However, more rigorous long-term studies with follow-up data for both intervention and – preferably active – comparator groups are needed to draw firmer conclusions. Non-inferiority trials may be particularly useful for evaluating whether STPP performs comparably to established treatments such as CBT [109], helping to assess its potential as a viable alternative for treating SAD.

The continued improvement observed at follow-up in some studies may partly reflect the slower change trajectory often associated with psychodynamic therapy [110], in which treatment effects emerge gradually over time. As such, one might argue that STPP is less dose-efficient compared to other short-term psychotherapy interventions – though such interpretation requires nuance. Literature suggests that short-term psychodynamic psychotherapy (STPP) can be effective for individuals with moderate symptom severity [111], while long-term PDT may be more appropriate for patients with more complex psychopathology, such as personality disorders or high comorbidity [112]. In line with this, the results of the present meta-analysis – which includes only STPP interventions – also indicate that relatively brief psychodynamic treatments may offer meaningful change for individuals with social anxiety disorder.

It is important to note that the current meta-analysis does not determine whether STPP benefits patients who have not responded to established first-line treatments such as CBT. The primary aim was to evaluate STPP’s potential efficacy as an alternative intervention, not its effectiveness in addressing treatment-resistant cases. To address that clinical question, future research should first consolidate efficacy findings and subsequently examine sequential treatment designs, such as cross-over trials where non-responders to one condition receive the alternative intervention [28].

A related consideration is that it remains unclear which therapeutical elements – in STPP as well as in other active treatment conditions – contributed to the observed effects. Clarifying mechanisms of change is not only of theoretical importance but also has clinical relevance [113]: deeper understanding of how specific components work may inform a more personalized approach to psychotherapy by helping match patients to interventions that best address their individual needs.

### Strengths, limitations and implications for future research

This review has several specific strengths that may contribute to the current understanding of the potential benefits of psychodynamic therapy for social anxiety. By focusing specifically on STPP for individuals with SAD, it offers a more detailed and disorder-specific synthesis than broader reviews that combine multiple diagnoses or therapeutic approaches. The inclusion of all available randomized controlled trials also ensures that the findings reflect the current state of the evidence as comprehensively as possible. Furthermore, by examining depressive symptoms alongside social anxiety outcomes, this meta-analysis provides a clinically relevant perspective. Despite its strengths, several limitations should be considered when interpreting the findings. First, although an extensive literature search was conducted, only 11 eligible studies were identified. This limited the statistical power, reducing the ability to detect moderator effects or draw firm conclusions about sources of heterogeneity. Although the meta-analysis increases overall statistical power, the relatively small size of several included trials may have limited the ability to detect subtle differences between STPP and comparison treatments. For the aims of the current meta-analysis – exploring the potential of PDT for SAD – the findings suggest that STPP may be an effective treatment for social anxiety. They provide a first step toward strengthening the empirical foundation of STPP, while highlighting the value of further research that includes clearly defined active comparators and larger samples.

Second, baseline social anxiety severity was assessed using different instruments, making the actual severity in each group uncertain. Although most LSAS-based studies clustered near the group mean (z < 0.23), some studies using other measures deviated substantially with z-scores > 1.00 [64, 81], suggesting variation in initial SAD severity. Ideally, this could have been examined as a moderator or adjusted for, but the limited number of included studies made such analyses infeasible.

Third, although quality of life was preregistered as an outcome, it could not be included in the analysis due to insufficient data, with only two studies reporting relevant measures. While symptom reduction is essential, outcomes such as interpersonal functioning or quality of life may offer a broader perspective on the real-world impact of interventions, especially for a condition like SAD that significantly affects social and occupational functioning. Future research would benefit from incorporating such outcomes to better capture meaningful change and assess the broader effects of treatment.

## Conclusion

This meta-analysis explored the potential of psychodynamic therapy as a treatment alternative for social anxiety disorder. While findings suggest that STPP is more effective than passive control conditions and close to comparable to active treatments – with a non-significant trend slightly favouring the latter – the limited number of available studies and substantial variability in study characteristics call for cautious interpretation. Similar effects on comorbid depressive symptoms and the maintenance of gains over time further support STPP as a promising, acceptable option in the treatment of SAD. However, the evidence base remains small, and several important questions - such as which patients are most likely to benefit and how STPP compares to other treatments in the long term - remain unanswered. Therefore, rather than offering definitive conclusions, this meta-analysis provides a comprehensive overview of the available evidence and points to important areas where further research is both necessary and promising. Future studies should aim to strengthen the empirical foundation by comparing STPP to other treatments in adequately powered trials, examining long-term outcomes, and clarifying the mechanisms underlying change.

## Supplementary Information

Supplementary Material 1.

Supplementary Material 2.

## Data Availability

The datasets and supplementary materials supporting the conclusions of this article are available in the Zenodo repository (DOI: 10.5281/zenodo.17975511). Supplementary files are also provided with the published article.
