# Supplementary material for: Short-term psychodynamic psychotherapy for social anxiety disorder: a meta-analysis of randomized controlled trials
Source: BMC Psychol. 2026 Mar 5;14:484. doi: 10.1186/s40359-026-04306-x (PMC13064217; doi:10.1186/s40359-026-04306-x)
Supplement: Supplementary file 2 — Supplementary Material 2. [file 40359_2026_4306_MOESM2_ESM.docx]

**Appendices**

**Appendix A** Database Searches 2

**Appendix B** Trim-and-Fill Plots Main Analyses 3

**Appendix C** Forest Plot Sensitivity Analysis SAD Outcome 4

**Appendix D** Forest Plot Depression Outcome 5

**Appendix E** PQRS Ratings 6

**Appendix F** Risk of Bias Assessment 7

**Appendix A: Database Searches**

**Table 1**

| **Population** | “social anxiety disorder” OR “social phobia” OR “anxiety disorder” OR “generalized social anxiety” OR “social fear” OR “excessive shyness” |
| --- | --- |
| **Intervention** | “psychodynamic therapy” OR “PDT” OR “psychoanalytic therapy” OR “psychoanalysis” OR “short-term psychodynamic therapy” OR “long-term psychodynamic therapy” OR “dynamic psychotherapy” |
| **Comparisons** | “waiting list” OR “wait-list” OR “psychotherapy” OR “cognitive behavioral therapy” OR “CBT” OR “exposure therapy” OR “third-wave therapy” OR “mindfulness-based therapy” OR “acceptance and commitment therapy” OR “psychosocial treatment” OR “behavior therapy” OR “interpersonal therapy” OR “psychopharmacotherapy” OR “pharmacotherapy” OR “antidepressants” OR “SSRI” OR “SNRI” OR “medication” OR “placebo” OR “pill placebo” OR “care as usual” OR “treatment as usual” OR “TAU” OR “usual care” OR “control group” OR “randomized controlled trial” OR “RCT” |
| **Outcome** | “social anxiety symptoms” OR “social anxiety score” OR “social phobia scale” OR “Social Phobia and Anxiety Inventory” OR “SPAI” OR “Social Phobia Scale” OR “SPS” OR “Social Interaction Anxiety Scale” OR “SIAS” OR “Liebowitz Social Anxiety Scale” OR “LSAS” OR “Fear of Negative Evaluation Scale” OR “FNE” OR “Kutcher Generalized Social Anxiety Disorder Scale for Adolescents” OR “K-GSADS-A” OR “depression symptoms” OR “depression score” OR “GAD-7” OR “PHQ-9” OR “Beck Depression Inventory” OR “quality of life” OR “QoL” OR “mental health improvement” OR “psychological well-being” |

*Search String Used in EBSCOhost and ProQuest*

**Table 2**

| **Population** | ("social anxiety disorder" OR "social phobia" OR "generalized social anxiety") |
| --- | --- |
| **Intervention** | ("psychodynamic therapy" OR "psychoanalytic therapy" OR "short-term psychodynamic therapy" OR "dynamic psychotherapy") |
| **Comparisons** | ("randomized controlled trial" OR RCT OR "control group") |
| **Outcome** | ("Social Phobia Scale" OR SIAS OR LSAS OR "Fear of Negative Evaluation Scale") ("quality of life" OR QoL OR "psychological well-being" OR "mental health improvement" OR PHQ-9 OR BDI) |

*Search String Google Scholar*

**Appendix B: Trim-and-Fill Plots Main Analyses**

**Figure 1**

*Trim-and-Fill Funnel Plot of SAD Effect Sizes Including Passive Controls and Imputed Studies*


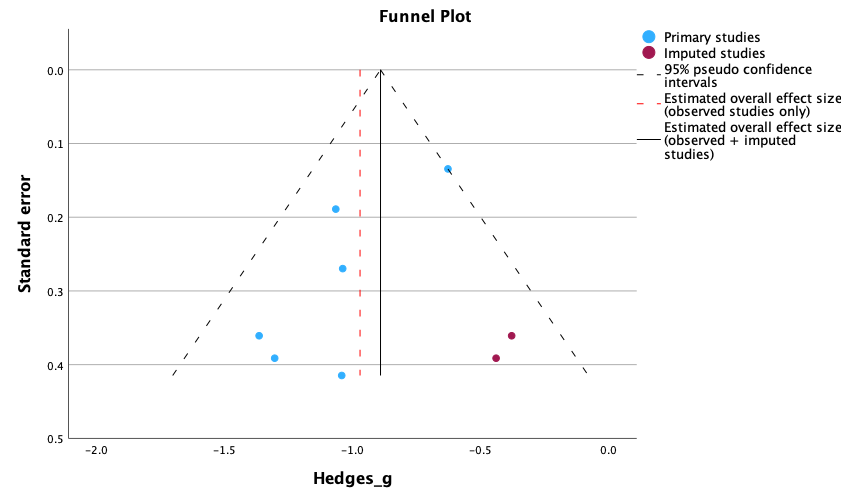


**Figure 2**

*Trim-and-Fill Funnel Plot of SAD Effect Sizes Including Active Controls and Imputed Studies*
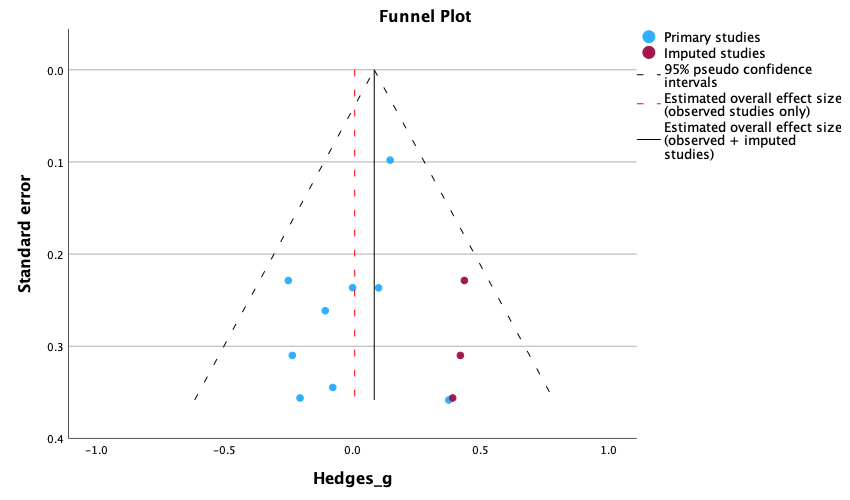


**Appendix C: Forest Plot Sensitivity Analysis SAD Outcome**

**Figure 1**

**
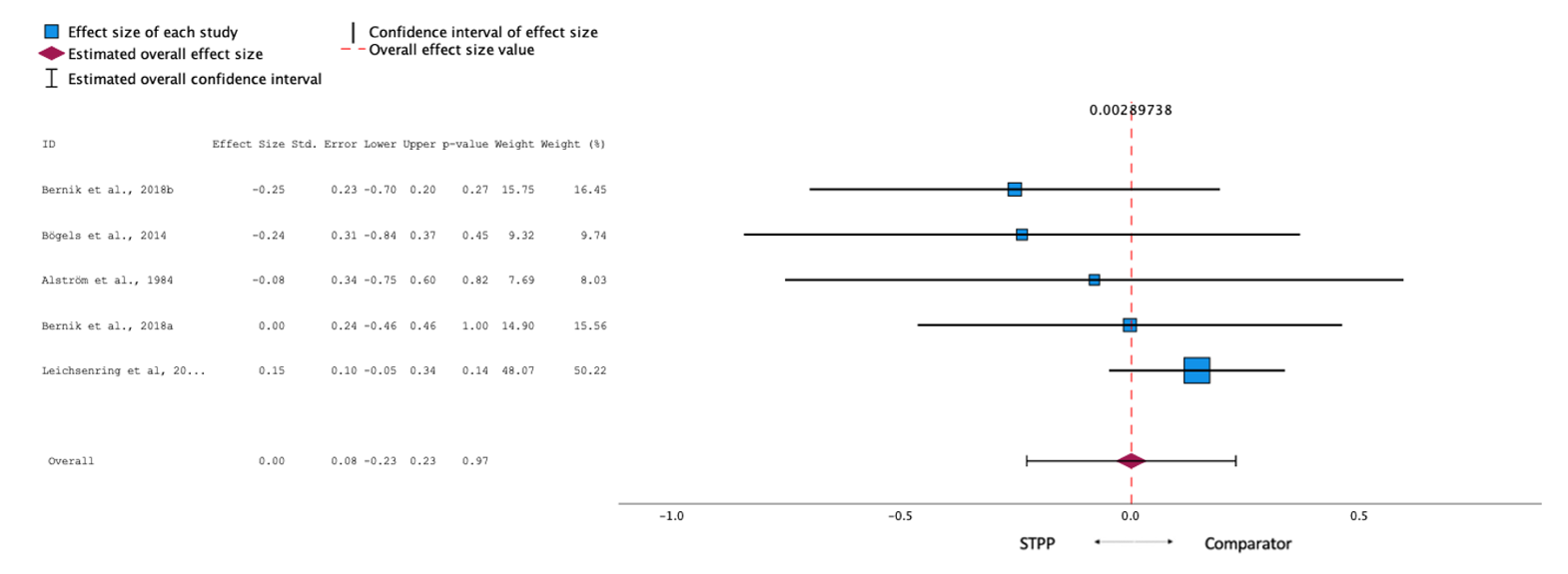
***Forest Plot of Between-Group Effect Sizes of STPP Compared to CBT*

**Appendix D: Forest Plot Depression Outcome**

**Figure 1**


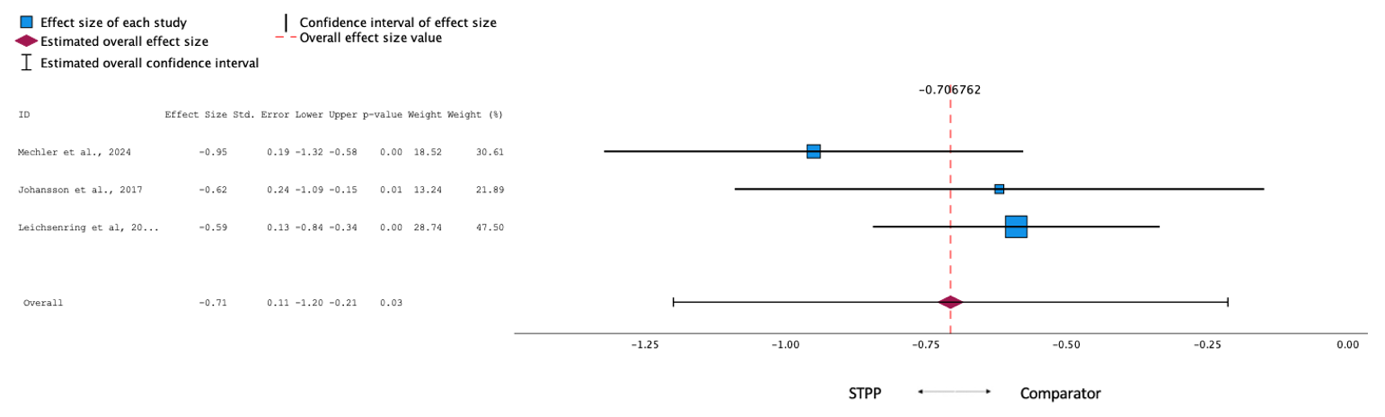
*Forest Plot of Between-Group Effect Sizes of STPP Compared to Waitlist*

**Figure 2**


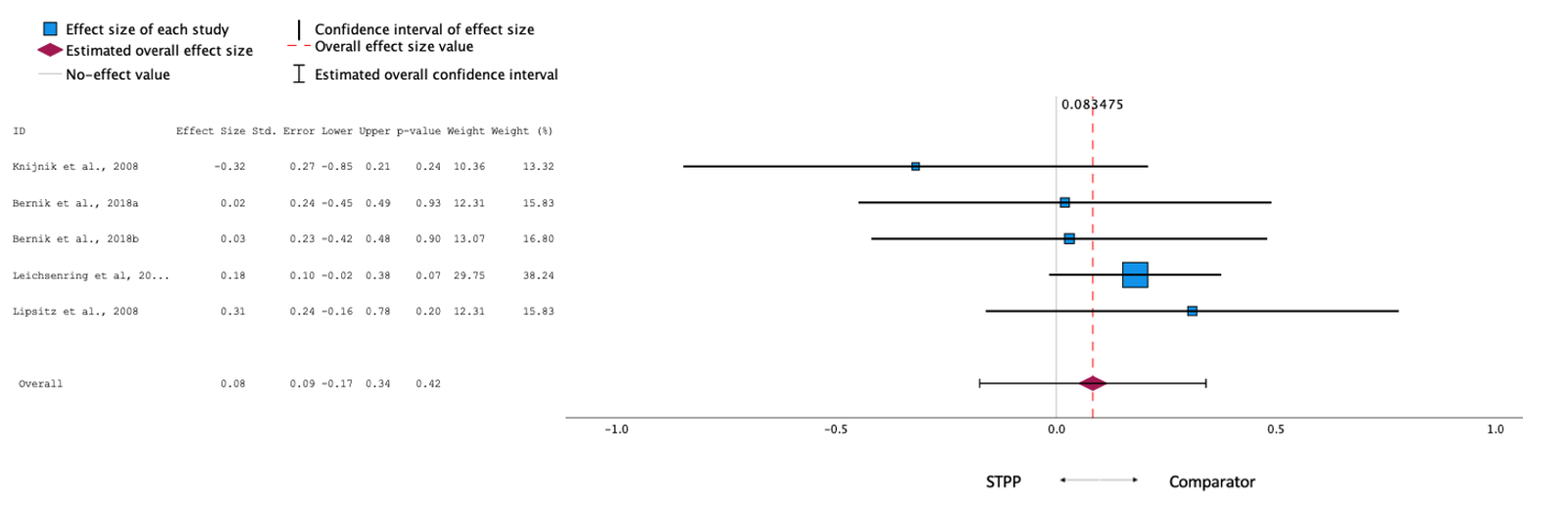
*Forest Plot of Between-Group Effect Sizes of STPP Compared to Active Comparisons*

**Appendix E: PQRS Ratings**

**Table 1**

| **Author (year)** | **Total score rater 1** | **Total score rater 2** | **Omnibus score rater 1** | **Omnibus score rater 2** | **Mean total score** | **Mean omnibus score** |
| --- | --- | --- | --- | --- | --- | --- |
| Alström et al., 1984 | 17 | 18 | 2 | 2 | 17.5 | 2 |
| Bernik et al., 2018 | 26 | 25 | 4 | 4 | 25.5 | 4 |
| Bögels et al., 2014 | 33 | 33 | 5 | 5 | 33 | 5 |
| Johansson et al., 2017 | 36 | 37 | 6 | 6 | 36.5 | 6 |
| Knijnik et al., 2004 | 21 | 20 | 3 | 3 | 20.5 | 3 |
| Knijnik et al., 2008 | 29 | 28 | 5 | 4 | 28.5 | 4.5 |
| Leichsenring et al., 2013/2014 | 44 | 45 | 7 | 7 | 44.5 | 7 |
| Lipsitz et al., 2008 | 29 | 29 | 4 | 4 | 29 | 4 |
| Mechler et al., 2024 | 33 | 32 | 5 | 5 | 32.5 | 5 |
| Moghadam et al., 2015 | 16 | 18 | 3 | 3 | 17 | 3 |
| Rahmani et al., 2020 | 17 | 17 | 2 | 2 | 17 | 2 |

*Individual PQRS-Ratings and Mean Scores*

*Note.* Excellent inter-rater reliability was found, with

**Total PQRS score**: ICC (2,2) = 0.997, 95% CI [0.989, 0.999], *F*(10, 10.2) = 294, p < .001.

**Omnibus score**: ICC (2,2) = 0.991, 95% CI [0.968, 0.998], *F*(10, 11) = 110, p < .001.

**Appendix F: Risk of Bias assessment**

The following criteria of the Cochrane Risk of Bias tool (Sterne et al., 2019) were assessed:

- Was the allocation sequence random?
- Was the allocation sequence concealed until participants were enrolled and assigned to interventions?
- Were outcome assessors aware of the intervention received by study participants? Or self-report measures only
- Dealing with incomplete outcome data (use of intention-to-treat-analysis)

**Table 1**

| Study | Random sequence  generation | Allocation concealment | Blinding of assessors or self-report measures only | Complete data (Intention-to-  treat-analysis) | Number of  criteria fulfilled |
| --- | --- | --- | --- | --- | --- |
| Alström et al., 1984 | ? | ? | - | ? | 0 |
| Bernik et al., 2018 | ? | ? | - | + | 1 |
| Bögels et al., 2014 | + | - | + | + | 3 |
| Johansson et al., 2017* | + | + | + | + | 4 |
| Knijnik et al., 2004 | ? | ? | + | - | 1 |
| Knijnik et al., 2008 | + | ? | + | + | 3 |
| Leichsenring et al., 2013/2014 | + | + | + | ? | 3 |
| Lipsitz et al., 2008 | + | ? | + | - | 2 |
| Mechler et al., 2024* | + | + | + | + | 4 |
| Moghadam et al., 2015 | ? | ? | - | - | 0 |
| Rahmani et al., 2020* | ? | ? | + | - | 1 |

*Ratings of Four Criteria of the Cochrane Risk of Bias Tool*

*Note.* low (+), unclear (?) and high (-)

* self-report only
